# A Manual of Auscultation and Percussion

**Published:** 1845-12

**Authors:** 


*A Manual of Auscultation and Percussion.* By M. Barth, Agregi to the Faculty of Medicine of Paris, &c. &c.; and M. Henry Roger, Physician to the Bureau Central of the Parisian Hospitals, &c. &c. Translated with additions by Francis G. Smith, M. D., Lecturer on Physiology, in the Philadelphia Medical Association, &c., Philadelphia: Lindsay & Blakiston, 1845. 12mo. pp. 160.

We are glad to see this little book, and so will be many a student, and perhaps many a physician, who has tried in vain to obtain correct views of Auscultation and Percussion from the verbose and contradictory narrations and descriptions scattered through the writings of pathologists, who seem to vie with each other, in making the subject as intricate and unintelligible as possible.

Unfortunately, the method of examination in question, affords from its very nature, extraordinary opportunities for deception, both undesigned, as affecting the mind of the explorer, and intentional as conveying false intelligence to others, or creating undue estimates of the physician's sagacity and wisdom. Many honest men have been so disgusted with the humbuggery which has been practiced by the artful, under the name of auscultation, that they have repudiated the practice altogether, as a mere trick, to get reputation without deserving it, and many more have found the printed lessons on the subject so unintelligible, as to cause them to abandon in despair all further attempts to understand the mode of physical exploration. Indeed it is hard to convey ideas of sounds, by words. Who would have an idea of the noise of thunder, by merely hearing the word pronounced, much less could any one convey to a pupil proper information of the different sounds of a piano, without exhibition of the instrument itself. How long would it take a pupil to learn to tune a guitar without seeing one, or touching its strings? Yet to do these things would be easy, compared with the difficulty of conveying to the medical student, accurate ideas of the obscure sounds of the chest, by terms descriptive of them.

Auscultation and percussion, have been taught to death. Every lecturer and writer, seems to have thought it necessary to describe new sounds or give new names to old ones, until the pectoral gamut is too rich for any practical use.

We believe that much information may be obtained from auscultation and percussion. There are certain leading sounds characteristic of important diseased conditions with which the student may easily acquaint himself. For nicer discrimination of more obscure pathological states, he must depend upon his own experience and observation.

We commend this little Manual to the student and to the physician. It comes from a proper source, and is well translated.

